# In the line of fire: Debris throwing by wild octopuses

**DOI:** 10.1371/journal.pone.0276482

**Published:** 2022-11-09

**Authors:** Peter Godfrey-Smith, David Scheel, Stephanie Chancellor, Stefan Linquist, Matthew Lawrence

**Affiliations:** 1 School of History and Philosophy of Science, University of Sydney, Sydney, NSW, Australia; 2 Institute of Culture & Environment, Alaska Pacific University, University Drive, Anchorage, AK, United States of America; 3 Department of Biological Sciences, University of Illinois at Chicago, Chicago, IL, United States of America; 4 Philosophy Department, University of Guelph, Guelph, ON, Canada; 5 Huskisson, NSW, Australia; University of Ferrara Department of Life Sciences and Biotechnology: Universita degli Studi di Ferrara Dipartimento di Scienze della Vita e Biotecnologie, ITALY

## Abstract

Wild *Octopus tetricus* frequently propel shells, silt, and algae through the water by releasing these materials from their arms while creating a forceful jet from the siphon held under the arm web. These "throws" occur in several contexts at a site in Jervis Bay, Australia, including in interactions with other octopuses. Material thrown in interactive contexts frequently hits other octopuses. Some throws appear to be targeted on other individuals, as suggested by several kinds of evidence: Throws in interactive contexts were more vigorous than others, and more often used silt, rather than shells or algae. High vigor throws were more often accompanied by uniform or dark body patterns than other throws. Some throws were directed differently from beneath the arms and such throws were more likely to hit other octopuses. Throwing at other individuals in the same population, as apparently seen in these octopuses, is a rare form of nonhuman projectile use, previously seen only in some social mammals.

## Introduction

The throwing of objects is an uncommon behavior in animals. A throw can be distinguished from other phenomena by the ballistic motion of a manipulable object or material, where "ballistic" describes free motion with momentum. Throwing with aiming has sometimes been seen as distinctively human, and it probably does have an important role in hominin evolution [1,2]. But throwing at a target has also been observed in some non-human primates (especially chimps and capuchins), elephants, mongooses, and birds [3–7]. Related behaviors include the flicking of irritating hairs at threats by spiders, the squirting of water through the air at prey by archerfish, and the swinging of silk threads at prey by some bolas spiders [8–10].

The processing of prey by various predators, such as dolphins, features thrashing and tossing above the water, and dolphins have also been observed tossing objects, sometimes between individuals, in play [11]. Antlion larvae fling sand when prey is attempting to escape from their conical traps, and these throws are oriented towards the direction of the prey [12], though the throws may function as much to destabilize the walls of the trap as to disrupt behavior of the prey itself.

The determination of targeting in a throw or related behavior poses challenges (see Discussion). When present, directed throwing can target prey or hard-shelled food (seen in birds and mongooses), threats, or otherwise problematic animals of other species (elephants, various primates). More rarely, directed throwing targets other individuals of the same population (chimps, capuchins, dolphins; see [3,6,11]). Throwing of this last kind is a form of tool use oriented to a conspecific; a projectile can be an agonistic, or communicative, social tool.

Octopuses as a rule are anti-social. They hunt alone, may fight on encounter, and can cannibalize each other [13–15]. However, recent work with octopuses has shown tolerance, signal use and other conspecific-directed behaviors in some species [16,17]. Octopuses are also dextrous manipulators of objects. Octopus den-building and maintenance includes the use of diverse materials [18,19]. Veined octopuses (*Amphioctopus marginatus*) carry shelter in the form of nested coconut shell halves that are then reassembled [20].

The gloomy octopus (*Octopus tetricus* Gould, 1852) is a medium-sized benthic octopus common in temperate waters around Australia and New Zealand, that have been studied at two high-density sites in Jervis Bay, Australia where interactions are common [21,22,26]. Food is locally abundant and outside the site, suitable shelter appears scarce, leading to proximity of octopuses to each other within a sharply limited patch of suitable habitat. Here we provide the first report for any octopus species of a behavior frequently seen at these aggregations: the throwing or projection of debris, both in social interactions and in other contexts. Ballistic motion of manipulable objects is possible through water, albeit against greater resistance than through air. These throws by *O*. *tetricus* sometimes hit other octopuses. We compare this behavior– a jet-propelled throw–to other ways that octopuses manipulate objects and use their jets, examine the roles this behavior has in conspecific interactions and other contexts, and investigate the hypothesis that some throws may be targeted on other octopuses. As we do not assume that the presence of a hit itself shows targeting (hits may be accidental), we attempt to assess whether additional systematic differences exist between throws that hit other octopuses and throws that do not.

## Methods

We studied *Octopus tetricus* at a field site at 17 m depth in the southern part of Jervis Bay, NSW, Australia (for details, see [21,22]). We collected behavioral data at this site using stationary video cameras (GoPro brand, various models) left on tripods at the site on various occasions from 2011 to 2018, in a combination of recreational diving and research diving. Having informally observed what appeared to be "shell throws" on several occasions, we examined our video records for behaviors of this kind. For each observation period we noted the presence or absence of jet-propelled throwing. A fortuitous combination of circumstances in 2015 gave rise to an unusually good set of data: water clarity was good; many octopuses were present; a fish nipped and scarred an active male, providing a distinct individual mark; and we were able to use natural markings to distinguish two females (S1 File). These circumstances made the 2015 data suited to examine relationships between throw contexts and throw properties (such as thrower sex and ID, body pattern display, throw vigor and thrown materials). We tabulated every 2015 recorded interaction and throw by reviewing and scoring all video from that entire observation period, comprised of one full day (approximately underwater first light to sunset; see S1 File) and two adjacent part days (the prior afternoon and following morning) of daylight filming in January. We also report data from a section of a period of filming from a single day in December 2016. This section of video was marked by notable examples of the patterns we describe using the 2015 data.

Tracking of individuals is difficult at this site, as octopuses frequently enter and leave the area of video coverage. Much of our analysis does not depend on identifying individuals (see below); however, we report some data dependent on individual recognition, where permitted by our data. We used a conservative approach to the re-identification of individuals engaged in throws, and did not take general similarity in appearance and behavior as sufficient for re-identification. A few individuals bore distinct markings allowing re-identification (see S1 File).

We assigned sexes to octopuses using behavioral criteria, as strict anatomical identification would require considerable interference with octopus behavior (see S1 File). We scored thrower body pattern as mottled, uniform-dark, uniform-intermediate, and pale with dark eyes (PDE, probably a disruptive pattern [23,24] though usually only the top of the body could be seen). We categorized the materials octopuses propelled as *shells*, *silt*, and *algae*, and scored each throw according to its main material (S1 File). Throws containing similar amounts of two materials were scored as mixed, and contributed 50% to each of the totals for those materials. We qualitatively scored throws as high, medium, or low vigor (SI File). We also identified the overall behavioral context of each throw, independent of potential measures of aiming. *Eating* throws discarded the remains of a meal after foraging and eating (the duration of which could exceed 30 minutes). *Den maintenance* throws were preceded, within 2 minutes, by rearranging or excavation of materials from in or around the den. Interactive throws were preceded by an interaction, within 2 minutes, with another octopus. Interactions included fights, mating attempts, and approaches or reaches with one or more arms toward another octopus, followed by an apparent reaction by another octopus (ranging from alerting to redirection to physical contact). Contexts were not mutually exclusive, as, for example, an octopus could interact with another octopus while cleaning its den; we scored these throws as mixed context. Other cases were scored as "no context," when none of these indicators were present. In a small number of cases (N = 4) that were difficult to score, events outside the 2 minute window were taken into consideration or events within it set aside (see S1 File).

We defined a throw as a ‘hit’ if another octopus interrupted the motion of the thrown materials (shells or algae contacted the octopus) or when thrown silt enveloped part of another octopus. Statistical tests of contingency (Results and S1 File) were used to determine whether various properties of throws varied across contexts and whether throws that hit other octopuses differed in other respects from those that did not.

This study comprised unobtrusive observation only of undisturbed wild non-protected invertebrate animals that were not manipulated in any way, with the exception of occasional nondisruptive interaction with octopuses during recreational dives. The 2016 data collection was approved by the Alaska Pacific University Institutional Review Board and the University of Sydney Animal Ethics Committee.

## Results

The 2015 data comprises 21 hours 7 mins of video in total, of which 13 hours 29 minutes were recorded on the middle day. In that 2015 data, from four to as many as eight individuals were visible at a time at the site and engaged in 1543 interactions. We estimate, based on diver counts of numbers both on and around the site during the period of data collection, that the total number of individual octopuses present in the 2015 data was around 10. We observed a maximum instantaneous count of eight octopuses, probably (see S1 File) three males and five females. During many periods, there was a particularly active male present (the "most active male," below), plus several females, and often an additional peripheral male. The most active male interfered with approaching and departing octopuses to various degrees.

The average hourly high count of octopuses present (the maximum for each hour, averaged) on the day with the largest sampling was 6.3 individuals (s.d. = 0.89). Interactions were frequent; from 11 up to 234 per hour, average of 73 (s.d. 60.96; see S1 File). These included fights of various levels of intensity, matings, and also approaches or reaches from one octopus to another, who then reacted (with color or posture change, another reach, ducking, retreat, etc).

**Fig 1:**
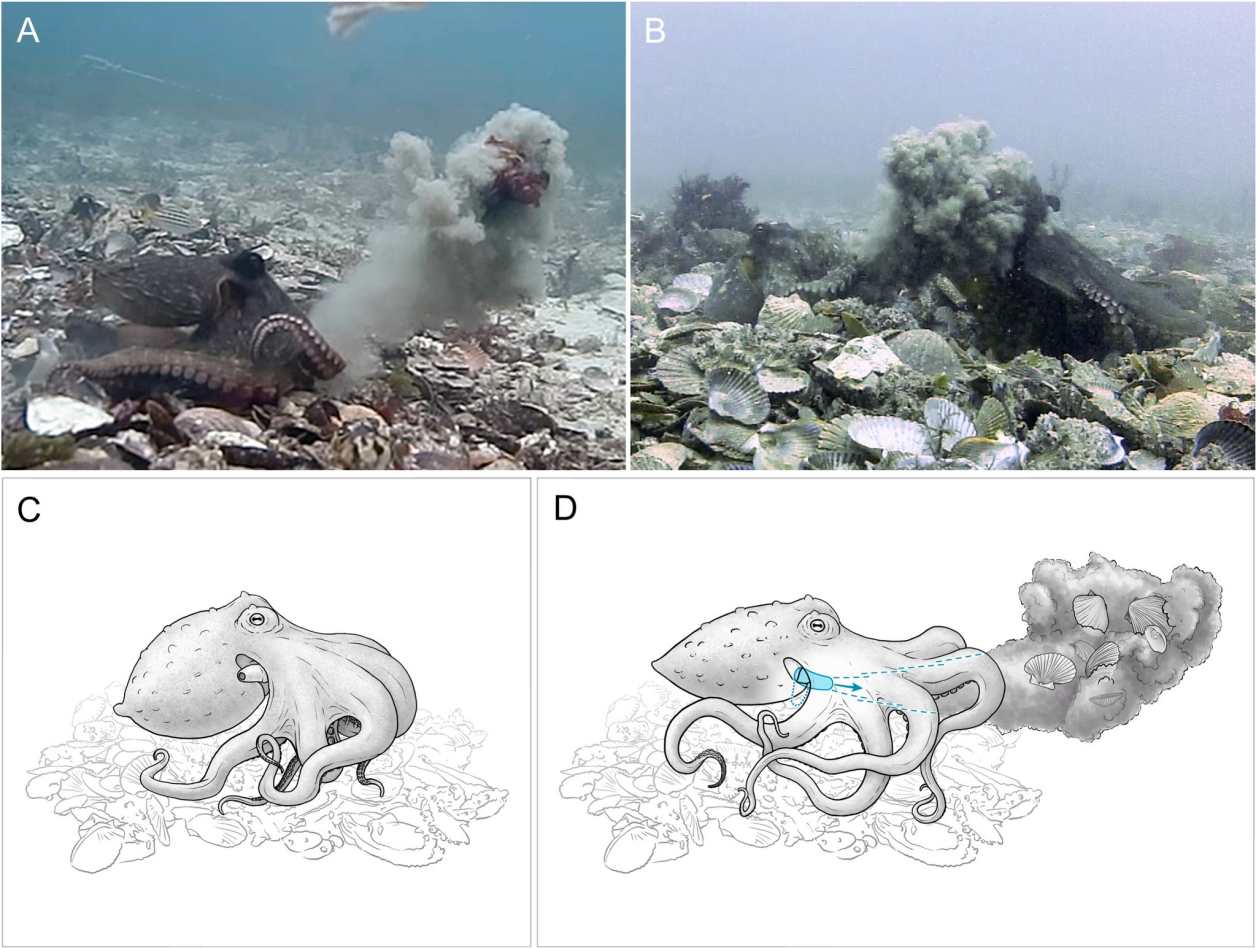
Debris throwing by *Octopus tetricus* in the wild: Panel A—Octopus (left) projects silt and kelp through the water (from video by Peter Godfrey-Smith); B–an octopus (right) is hit by a cloud of silt projected through the water by a throwing octopus (left; see SI for video of this event); C, D The mechanics of throwing behavior, C–shells, silt, algae or some mixture is held in the arms preparatory to the throw, mantle is inflated preparatory to ventilation during the throw, siphon at this stage may still be visible in its usual position projecting from the gill slit above the arm crown; D–siphon is brought down over rear arm and under the web and arm crown between the rear arm pair (arms R4 and L4), and water is forcibly expelled through the siphon, with contraction of the mantle, as held debris is released, projecting debris through the water column. Illustrations by Rebecca Gelernter.

A conspicuous behavior observed in the video data involved the coordinated use of arms, web, and siphon, by which material was projected through the water column (Fig 1A and 1B; S1 Videos). In this behavior, octopuses gather material in their arms, hold it in the arms and web, and then use the siphon to expel the material under pressure. We could seldom view all mechanics of these actions on a video, but typical cases proceed as follows: after the gathering of material, commonly from inside the den, the siphon is brought under the web of the octopus’ arms, by bringing it between arms L4 and R4 (the two rear-most arms). Water is then expelled forcibly as the material held is released (see Fig 1C and 1D). The material is ejected from between arms L1 and R1 (the two front-most arms) in the majority of throws, but in some cases the material was ejected between other adjacent pairs of arms, or directly under one arm (see S1 Videos, especially videos 2 and 5). These jets may be strong enough to propel material several body-lengths from the animal in still water, or so weak that the material falls almost directly in front of the animal. In some cases the projected material hits another octopus, or another object (a fish or a camera).

We interpret these behaviors as *throws*. The behavior could equally be described as an arm-guided release of material propelled (in every case except one that we observed; see below) by force arising from the jet. The previously described "Remove" behavior [25] broadly construed– using the jet on material regardless of whether it is gathered and held– includes throws in our sense, along with behaviors in which a water jet is directed on an object that was not held. A clear case of a throw in our sense requires a water blast from the siphon that is directed at and simultaneous with release of material held in the arms, requiring that the siphon move into an unusual position below the arm web.

Throwing by octopuses occurs repeatedly at this site and also occurs at another site [26]. We recorded throwing as early as 2011 ([24], see supplemental video C) and throwing has been recorded at at least one of these sites in five of six years during visits of various durations from 2013 to 2018. In this report, we closely examine throws in data collection periods during 2015 and 2016.

Throws in this sense do shade into other behaviors, as the gathering and holding of material can be minimal. We include in our dataset here borderline cases where a jet was used from below but the gathering of material was minimal (these cases comprise about 11% of the total; see S1 File) along with one unusual case where a shell was, at least in part, flung by straightening an arm, and hit another octopus. For brevity, we refer to all items in the data set as "throws," where this should be understood to include the borderline cases. We do not count cases where material propelled is clearly not held, held materials are not released, and/or the siphon is directed on material while the siphon is above the arm web.

### Throws by different individuals and sexes

We observed N = 102 of these throw behaviors in the 2015 video data. Two females accounted for the majority (66%) of the throws, but likely half or more of individuals present threw at least occasionally, both sexes threw, and octopuses threw from locations around the site (see S1 File). There were 90 throws by females and eleven by males, a ratio of 8.9:1. The female to male sex ratio at the site probably ranged from around 1.7:1 to around 5:1. This ratio varied continually as octopuses came and went, and could not be precisely determined at each moment.

### Context of behaviors, and materials thrown

Over half of all throws (53%) occurred in interactive contexts (36% interactive only, 17% interactive-mixed, see Methods and S1 File); 32% occurred during den-cleaning alone, 8% after eating, and 8% without apparent context (total > 100% due to rounding).

**Fig 2:**
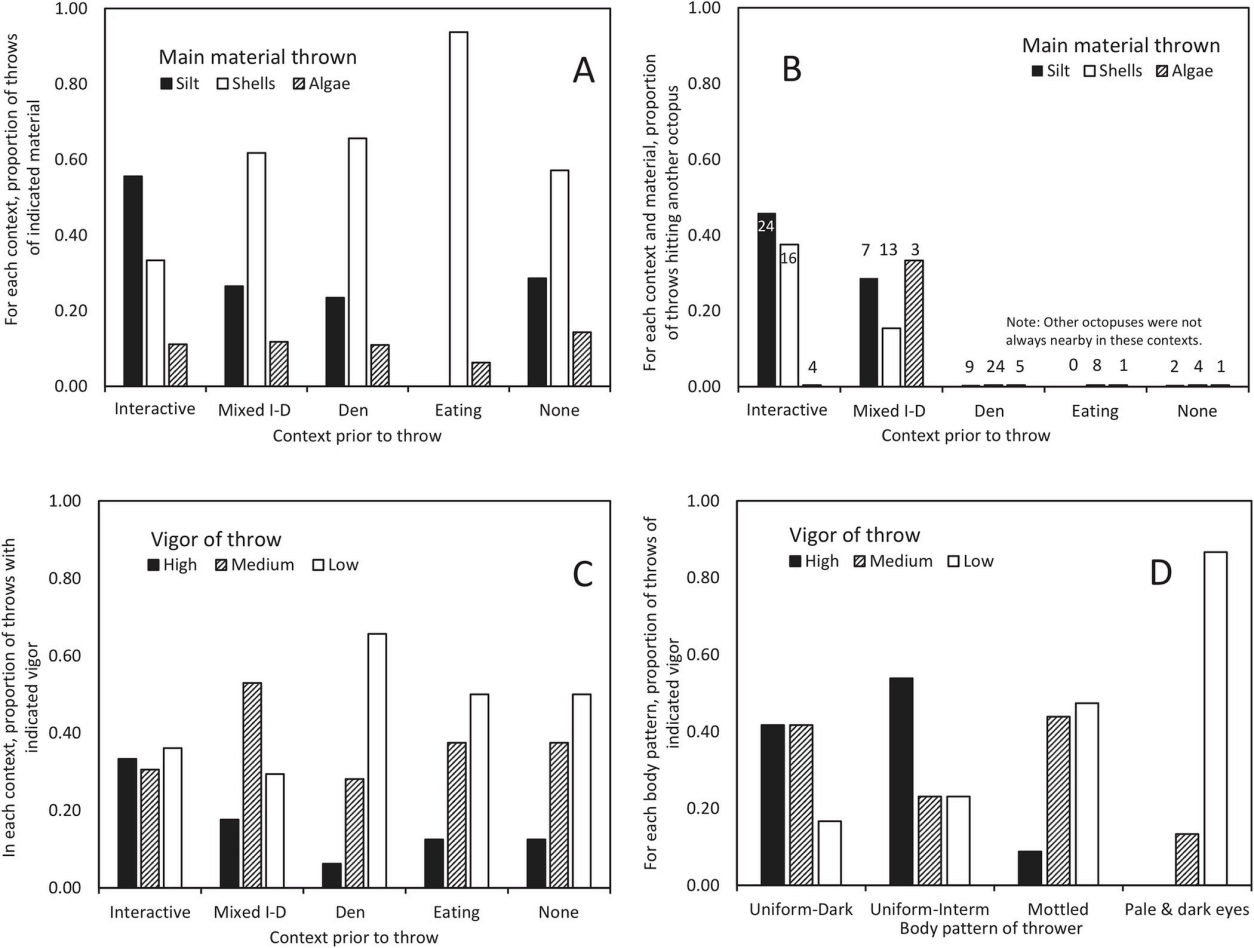
Throwing behavior by *Octopus tetricus* in the wild: Panel A, B of different materials by context (“mixed I-D” indicates both interaction and den maintenance occurred prior to the throw, see text for details), and C, D thrown with varying vigor. Panel A–the proportion of all throws; B–the proportion of throws that hit another octopus (numbers above columns indicate sample size of throws of that context and material); C–the proportion of throws by context; and D–by body pattern displayed during the throw by the thrower.

Interactive throws differed from other contexts in the materials thrown. Shells were thrown most often in general and algae least: N = 55 shell throws, 35 silt throws, and 11 algae throws (counting mixed-material throws as half a throw of each of the two materials). In interactive contexts, octopuses threw silt significantly more often than in other contexts, whereas they threw shells more often in den cleaning contexts (Fig 2A and 2B. Chi-square test: N = 76, χ^2^ = 9.26, df = 2, p < 0.01. Only interactive, mixed, and den cleaning contexts were considered due to sample size).

Significantly more interactive throws were high vigor and den cleaning throws were low vigor (Fig 2C. Considering just throws in interactive, mixed or den contexts (those with the greatest sample size), Chi-square test: N = 85, χ ^2^ = 12.98, df = 4, p = 0.011). Overall, however, low vigor throws were most common. (Low vigor: 48 of 102 throws (47%); medium, 35 throws (34%); high, 19 throws (19%)).

Octopuses that displayed dark or medium uniform color at the moment of the throw threw significantly more often with high vigor, while those displaying a "pale and dark eyes" pattern threw more often with low vigor, (chi-square test: N = 102, χ^2^ = 29.7, df = 4, p < 0.0001). Those displaying a mottled pattern also threw less often with high vigor. Vigor was also associated with the color of the thrower at the moment of throw (see Fig 2D). Earlier work at this site has found that darker colors are associated with more aggressive behaviors [25].

### Hits on other octopuses

The material thrown hit another octopus in 17 cases. These were 33% of the throws in interactive and mixed-interactive contexts. In two other cases, a throw hit a fish. Throws that hit another octopus tended to differ in several ways from throws that did not. These differences collectively suggest a degree of targeting. First, in a majority of all throws, the material was emitted from between the most frontal arms: L1 and R1. However, throws occasionally emitted material from between arms L1/L2, R1/R2, or directly beneath L1 or R1 (see S1 Videos, especially videos 2 and 5). A total of 14 throws out of 98 that could be assessed were "anomalous arm" throws of this kind. Anomalous arm throws were more likely to hit other octopuses than L1/R1 throws (p = 0.0145, Fisher’s exact test, 2-tailed). Of these 14 throws, 6 hit another octopus (43%), as compared to 11 hits from 84 throws (13%) of the L1/R1 type. In three cases of hits, a thrower also altered their body orientation towards another octopus, but these movements were very slight, and the effects of arm choice other than L1/R1 were more marked.

Second, we noted above that throws by octopuses displaying uniform body patterns (especially uniform dark patterns) were more often thrown with high vigor. Further, throws by octopuses displaying uniform body patterns also hit other octopuses significantly more often than those in other body patterns (p = 0.0021, Fisher’s exact test, 2-tailed). In addition, high vigor throws more frequently hit another octopus (7 of 19 high vigor throws were hits (37%); 6 of 34 medium vigor (18%) and 4 of 48 low vigor throws (8%)) (p = 0.017, Fisher’s exact test comparing high to the combined categories of medium and low vigor throws). High vigor throws had longer and often wider range that could explain more frequent hits without deliberate targeting.

Third, we noted above a significant tendency for throwers in interactive contexts to throw silt rather than other materials. A similar difference occurred among throws that hit another octopus. Throws solely or primarily of silt, within interactive or mixed-interactive contexts, hit another octopus 8 out of 19 times (42%; e.g., S1 Videos, videos 1 and 3). In contrast, throws solely or primarily of shells in these contexts hit another octopus 4 out of 16 times (25%). This difference was not statistically significant. In only one case did a throw of algae (along with shells) hit another octopus.

### Sequences of throws with large numbers of hits

At the end of the fully sampled day in 2015, and also in a December 2016 sampled period, particular sequences provided circumstantial evidence of deliberate targeting. In each case, the context, behavioral details, and high number of hits support a social role for throws. In the 2015 period, of approximately 60 minutes, a single female (T23F) threw 17 times, of which 9 were hits on other octopuses. Of those 9 throws, 8 were hits on a nearby likely female and 1 was a hit on the most active male. Several of these throws occurred in a context of intermittent reaches and grappling of arms between the thrower and the probable female in an adjacent den. In some cases, the other octopus raised an arm up between itself and the thrower, just before the throw, perhaps in recognition of the act in preparation. These preparatory arm raises were more marked in the later throws of that sequence. Two of the hits within this sequence were throws where material was emitted between arms L1/L2 rather than L1/R1 (e.g., S1 Videos, video 2). In these cases, had the material been projected from the L1/R1 position, it would apparently have missed the other octopus, as this choice affected the angle of the throw.

This period in the 2015 data is similar in some aspects to another sequence of throws between two octopuses with frequent hits, in December 2016. In this period of approximately 3 hours 40 mins, during which individuals can be reidentified based on onscreen continuity and marks, a single female threw material 10 times, with 5 of these hitting a male in an adjacent den, who attempted several times to mate with her. The male also threw material either once or twice during that period, once disposing of shell remains after eating (with the other case perhaps similar but unclear). All ten of the female’s throws were entirely or partly silt throws. In one hit, the female’s preparatory motions included a turn towards the male, and the thrown material emerged between arms R1/R2, bringing the male directly into the path of the throw. This sequence is also notable for the behaviors of the male who was frequently hit. In 4 cases the male ducked during the process of the throw itself, and over the course of the sequence, these movements occurred earlier in relation to the throw. In the first two cases, this male ducked after release of the throw; in the latter two, he ducked before the release, during preparatory motions by the thrower (see S1 Videos, video 3).

### Throws toward a camera

One other category of throws in the 2015 data shows some evidence of targeting, though not in a way directly related to octopus interactions. In 12 throws, material was thrown in the direction of a nearby stationary camera. Throws often had insufficient force to hit the camera. In three cases, however, the thrower advanced toward the camera before making the throw, or advanced obliquely and threw from one side (between arms R1 and R2) toward the camera. In two cases (one with the throw between R1/R2), the throw hit the camera tripod. Both of these hits occurred during a data-collection period of roughly 100 minutes when a camera was accidentally placed closer to octopus dens than usual. The usual camera distance is about a meter or slightly more from the nearest den, and this placement was closer by approximately a third of that distance. During this period, the octopus in the den closest to the camera threw material 6 times in the direction of the camera, out of 18 throws during that period, including the two hits.

## Discussion

The throwing of material by wild octopuses occurs frequently at our two study sites. These throws are achieved by gathering material and holding it in the arms, then expelling it under pressure. Force is not imparted by the arms, as in a human throw, but the arms organize the projection of material by the jet (Fig 1).

Earlier descriptions and videos have noted other octopus species using their jets to clear debris from a den area, but directing the jet under the arm-web in the way described here is unusual, especially given the octopus body layout. The throw behavior has not been noted in ethograms (eg., [25]) as distinct from using the jet on loose materials. However, similar behaviors in discarding meal remains away from a den have been seen in some other species in captivity (larger Pacific striped octopus and *Octopus cyanea*, DS, personal observation). While observations used in this paper were made at a site where octopuses coexist in close proximity, throws functioning in den-maintenance and removal of food waste may be expected to occur also in contexts with little or no interactions among octopuses.

Throws that hit other octopuses were minority of cases, but occurred fairly often. As noted above, showing targeting in a behavior of this kind poses challenges, and this problem has been handled in several ways in earlier work (see Table 1). We present several types of evidence that, at the site described here, interactive throws in some cases are targeted on other octopuses, and suggest that they function in the management of social interactions, including sexual interactions. As described in the Results, throws in interactive contexts and throws that hit other octopuses differed in several ways from other throws observed. Hits in many cases also occurred within sequences of interactions that featured ongoing mild aggression (arm probes and momentary grappling). The octopus who was hit often altered its behavior in anticipation or reaction to a throw; octopuses in the line of fire ducked, raised arms in the direction of the thrower, or paused, halted or redirected their movements.

**Table 1:** Representative reports of animal throwing and related behaviors that assert targeting, with sample sizes and the criteria for targeting used.

Species	Throws	Hits	Projectile	Apparent Target	Aiming criteria	Citation
Chimpanzee	N = 12	N = 1	Sticks, stones, leaves	Humans, baboons	Direction of throw	[27]
	Numerous occasions		Sticks, stones, other objects	Humans, baboons chimpanzees		[3]
Capuchin	N = 63	N = 10	Stones	Capuchins	Prior directed attention and hits	[6]
Elephant	Repeatedly	N = 0	Mud, soil, vegetation	Rhino	Direction of throw	[4]
Polar Bear	N = 12	Yes	Ice blocks, rock, pipe	Walrus, meat target	Direction of throws, improvement over time	[28]
Egyptian vulture	N~11	N = 1	Stones	Egg	None	[7]
Archer fish	N>1000	Up to 80%	Water	Artificial target	Direction of throw, flexibly adjusted for moving target	[9]
Antlions	N = 574	n/a	Sand	Prey	Direction of throw, including changes to orientation	[12]
Octopus tetricus	N = 102	N = 17	Shells, silt, algae	Octopuses	Direction of throw, hits, mechanics of throws that hit, other behavioral differences	This report

The case for a partially social role for throwing at this site is tempered by the fact that there are some things we have *not* seen. We have not seen an octopus who was hit by a throw "return fire" and throw back. We have not seen a hit directly initiating a fight, due to immediate retaliation by the target. The general effects of throws were difficult to assess, and we were hampered by the limited number of hits. Some throws in what appear to be fairly intense interactions were not directed at another octopus but into empty space.

Showing intention in a behavior is difficult in non-human animals [18]. A deflationary interpretation of the tendency of throws to hit other individuals might be offered as follows: If an octopus in an interactive setting throws debris in the course of den-cleaning or for other non-social reasons, but does so while attending to another octopus, hits may arise fortuitously because of the thrower’s orientation to the other individual. This interpretation might explain why hits were associated with uniform coloration, but would not explain the tendency for octopuses to use unusual arm arrangements in cases when they hit other octopuses. Neither would it explain the more common throwing of silt in interactive contexts. These and other features of the manner in which hits arise do suggest that some throws are intended to hit others.

Even if no intention to hit other octopuses lies behind these throws, they do have social effects in interactions between individuals at this site. Octopuses can thus definitely be added to the short list of animals who throw or propel objects, and provisionally added to the shorter list of those who direct their throws on other animals. If they are indeed targeted, these throws are directed at individuals of the same population, and octopuses then join a small collection of social mammals in this rare form of nonhuman throwing.

## Supporting information

S1 File Supporting information for "In the Line of Fire: Debris Throwing by Wild Octopuses". Further description of Methods, and additional Results.
(PDF)

S1 Videos Zip file with five videos of octopus throws. See S1 File for descriptions of each throw.
(ZIP)

S1 Data Xlsx file with data on each throw. (XLSX)
