# Supplementary material for: In the line of fire: Debris throwing by wild octopuses
Source: PLoS One. 2022 Nov 9;17(11):e0276482. doi: 10.1371/journal.pone.0276482 (PMC9645608; doi:10.1371/journal.pone.0276482)
Supplement: S1 File — Further description of Methods, and additional Results. (PDF) [file pone.0276482.s001.pdf]

Supporting Information for "In the Line of Fire: Debris Throwing by Wild Octopuses"

Peter Godfrey-Smith, David Scheel, Stephanie Chancellor, Stefan Linquist, Matthew Lawrence

I: Videos

All videos have had their colors adjusted to increase contrast and the visibility of behaviors. Color adjustment was assisted by Alina Bermingham.

Video 1: A throw by a female octopus (T1F) that hits a male attempting to mate with her. The material thrown is silt, vigor is high, and thrower's pattern is dark uniform. (2015 data, throw 27-14)

Video 2: A throw by a female octopus (T23F) that hits another likely female. This throw is part of the concentrated sequence of throws with a high rate of hits recorded at the end of the full day of sampling in the 2015 data. The throw is directed from underneath arm L1, rather than between L1 and R1, so it is one of the "anomalous arm" throws discussed in the main text. The material thrown is a combination of shells and silt, vigor is medium, and the thrower's pattern is mottled. (2015 data, throw 28-46)

Video 3: A throw by a female octopus, hitting a behavioral male. The material thrown is silt. The male ducks just before the material is released. (2016 data, throw 17-11)

Video 4: A throw by a female octopus disposing of fresh shells after eating. The octopus returned from a foraging trip 18 mins earlier and assumed a characteristic feeding posture. As the motion of the throw begins, another octopus reaches towards her and they touch as the shells are released. The context of this throw was scored as *Eating* despite the reach. The vigor was scored as high and the pattern as mottled. (2015 data, throw 27-19)

Video 5: A throw by a female octopus engaged in den maintainence. In the 2 minutes prior to the throw, the octopus several times was engaged in bringing up shells from inside her den. The throw is directed from between R1 and R2, so it is another "anomalous arm" throw. The throw was scored as medium vigor with a mottled pattern. (2015 data, throw 29-07)

II: Methods and Further Details of Results

1. Timing of data collection

2015 video data comprised of one full day and two adjacent part days (the prior afternoon and following morning).

27 Jan 2015: 12:12 to 17:31, 5 h 19 min

28 Jan 2015: 06:49 to 20:20, 13 h 31 min

29 Jan 2015: 08:05 to 10:22 , 2 h 17 min

Video data was collected for a full day on 27 Dec 2016; this paper reports only on the period between 10.10 am and 1.41 pm (3 h 41 min). A camera malfunction and less favorable water clarity conditions prevented an analysis of the 2016 data along the lines of the 2015 data. During the time period indicated above, a pair of octopuses could be consistently reidentified through a series of notable behaviors.

In consultation with the other authors, PGS and DLS scored behaviors from video, including throws.

During these and other data collection periods, either two or three cameras were usually in operation at different positions along the edge of the site, one meter or more from the nearest den, with large but incomplete overlaps in coverage, and usually with several dens in view. Due to technical problems or disruptive animal behaviors, some cameras were occasionally unable to collect data.

2. Categorization of behaviors

As noted in the main text, throws of the kind described in this paper shade into other behaviors, because the gathering and holding of material can be minimal. Some apparent throws are also made from positions partially inside a den, making observation of behavioral details difficult. We opted to include, rather than ignore, some borderline cases that only minimally met our definition, as very little material was projected, the force of projection was minimal, and/or gathering prior to the throw was minimal. There were 11 (approximately 11% of N=102) such borderline throws; thus they comprise a small portion of our sample.

Throws were scored qualitatively for vigor. High vigor throws propelled a mass of material comparable in size to the animal's body, and/or propelled it a distance comparable to the animal's body length (SI Video 1). Low vigor throws propelled a much smaller quantity of material – one or a few shells, for example – a distance that was a small fraction of the animal's body size. Medium vigor throws were intermediate between these (SI Video 2). Silt throws produced larger plumes than other thrown materials; we made allowance for this fact informally. Borderline cases described above were included in low vigor throws.

We categorize the materials octopuses threw as shells, silt, and algae. Shells were bivalve shells, usually scallops that comprised the bulk of the site substrate. Algae were unattached pieces of brown or green algae often but not always within reach of an octopus. Octopuses gathered silt within an excavation (often within the den occupied by the thrower). We scored each throw as "shells", "silt", "algae" or "mixed" according to its main material. As noted in the text, throws containing similar amounts of two materials were scored as mixed, and contributed 50% to each of the totals for those materials, while we discounted minor contributions - for example, small amounts of silt in shell throws.

We tested the hypothesis that the properties of throws (body patterns, materials, vigor, as above) were the same in each context using chi-square contingency tests. We used Fisher's exact tests for the hypothesis that throws that hit another octopus did not differ from those that did not hit in three properties (orientation, body patterns and vigor). We tested the

hypothesis that the distribution of throws among individuals was unbiased using binomial distributions.

3. Individual identification

A few individuals had markings that enable reidentification across breaks in onscreen continuity; these were the two most frequent female throwers – T1F, T23F – and, for part of the data collection period, T6M (see Figure S1). It was not possible to track all individuals over long periods, so we did not attempt to determine whether the elevated observed rates of throwing by some individuals may be due to them spending more time on screen. As discussed in the main text, a particular den was very often the site of throws, a finding not affected by difficulties in tracking individuals.

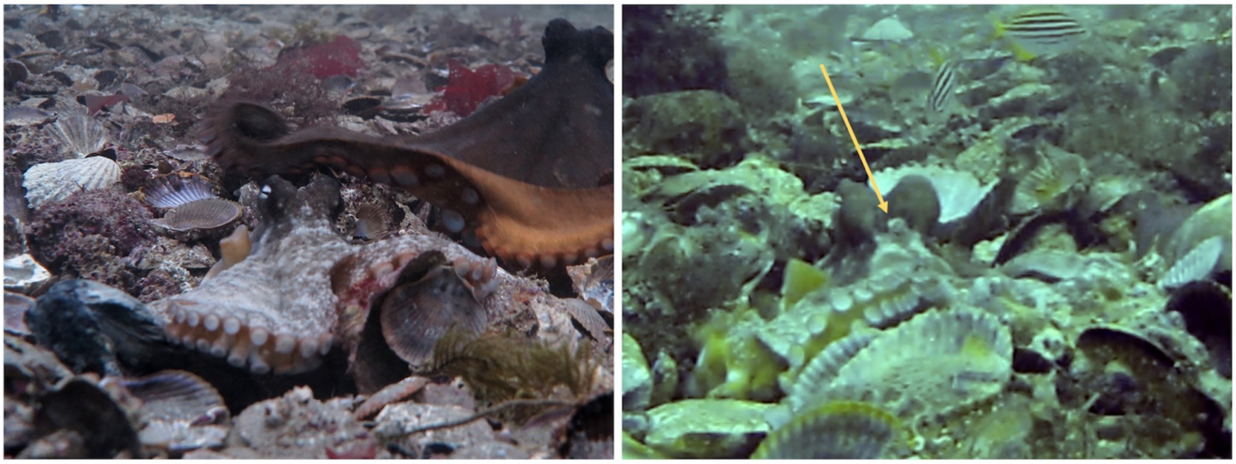

Figure S1: Individually recognizable markings below the eyes of two individuals, TIF (left) and T23F (right). For T1F, note the pale dot (papillae) below each eye higher than the frontal white spot and the prominent and almost unbroken horizontal white frontal bar below the frontal white spot. For T23F, note the arc-shaped or peanut-shaped white area comprising the upper part of the frontal white spot (indicated by the arrow). When the relevant body patterns were displayed by an octopus, these distinctive details were consistently noted on T1F and T23F respectively and were absent on other octopuses.

GoPro camera image on the right has been adjusted for color tone. Left image: photo by Peter Godfrey-Smith. Right image: from video data used in this paper.

Out of 102 throws in the 2015 data, these two females were responsible for 67 throws (41 by octopus T23F and 26 by T1F). For T23F singly and both together, this was significantly more than expected under a binomial distribution, assuming four or more potential throwers ($p=<0.001$). These two individuals were responsible for at least 66% of all throws, and likely another 5 throws when identifying marks were not visible on video to confirm a suspected ID. The most active male descibed in the main text may have been the next most frequent thrower, with 5 throws in total, though he could often only be provisionally reidentified until he was bitten by a fish midway through the recording period, leaving a visible scar.

The distribution of N=29 throws among other individuals is uncertain. We defined a *nominal* individual as an octopus continuously on-screen or with unique markings. The identification of nominal individuals assumes in effect that every break in onscreen continuity in the absence of distinguishing marks establishes a new individual. Thus the count of nominal individuals is higher than the number of octopuses likely visiting the site, based on counts in the field and identifications on video of octopuses entering and leaving the site. Using these identification criteria, there were 24 nominal individuals (including the three described above) who engaged in throws across the three days sampled in 2015 (7 females identified by mating behaviors, 7 by other behaviors (see SI above); 7 males identified by mating, 2 by other behaviors; one with no assigned sex).

A large number of throws were also made by octopuses while at a particular den. The most frequent throwers T1F and T23F each occupied this den at different times, as did other individuals briefly, and in total 59 throws (58%) originated at this location (57 throws by the two most frequent throwers). This den was closely flanked by other octopus dens, one at 20 cm, was close to a camera, and was frequently visited by other passing octopuses.

4. Determination of sex

As noted in the main text, we assigned sexes to octopuses using behavioral criteria, as strict anatomical identification would require considerable interference with octopus behavior. We identified most males and females by mating behaviors: males made mating attempts by extending arm R3; females accepted, or did not immediately rebuff, these mating attempts. A total of 89 of 102 throws were by individuals whose sex could be assigned through observation of mating attempts, based on nominal (conservative) identifications of individuals. In the remaining cases, we categorized an individual as behaviorally male or female based not on mating behaviors per se, but on other behaviors seen to be associated with male or female mating roles. In the case of males, these behaviors were: interfering with or blocking departure attempts by behavioral females; attending to but not opposing returns from off-site by behavioral females; aggression towards other likely males. In some cases, we used enlarged suckers as an anatomical indicator of male sex. In the case of females, these behaviors were avoiding aggression with behavioral males; being interfered with or blocked on departure attempts; being attended to and allowed to approach on return from offsite by behavioral males.

5. Contexts of behaviors

As described in the main text (Methods), we distinguished three contexts in which throws occurred. N=101 throws were scored for their context in this way (with one throw at the start of sampling not scored because the two minutes preceding the throw were not recorded.) Some throws were difficult to score for context and handled in a way that included consideration of factors outside the 2 minute window for interaction, or set aside events within it. In one case, a lengthy quiet mating included a clear eating throw by the female, and we scored this not as mixed, but as an eating throw. In a second case, no indicators of context occurred until after the throw was in process, 5 seconds before release, at which moment another octopus arrived, probably not influencing the throw; this we scored as no-context. In a third case, no relevant behaviors occurred within the 2 minute window but events outside it suggested a den-cleaning context. In a fourth case (see video 4 of SI), during the initial motions of a throw disposing of fresh prey remains after foraging, another octopus reached towards the thrower; this was categorized as an eating

throw (SI Video 4). Two cases were also ambiguous because, as discussed in the main text, an octopus may have been responding in part to the presence of a fixed camera on a tripod within a meter of its den. Based on other behaviors, both were scored as den-cleaning throws. A total of six cases of 101 were thus ambiguous in these ways.

6. Timing of throws

On the day in 2015 with 13.5 hours of sampling, throws occurred throughout daylight hours but increased in frequency around dusk (see figure S2). This timing coincided with the largest number of interactions in an hour, as well as the largest count of octopuses simultaneously in frame. At dusk, octopuses remained active until the video became too dark to evaluate.

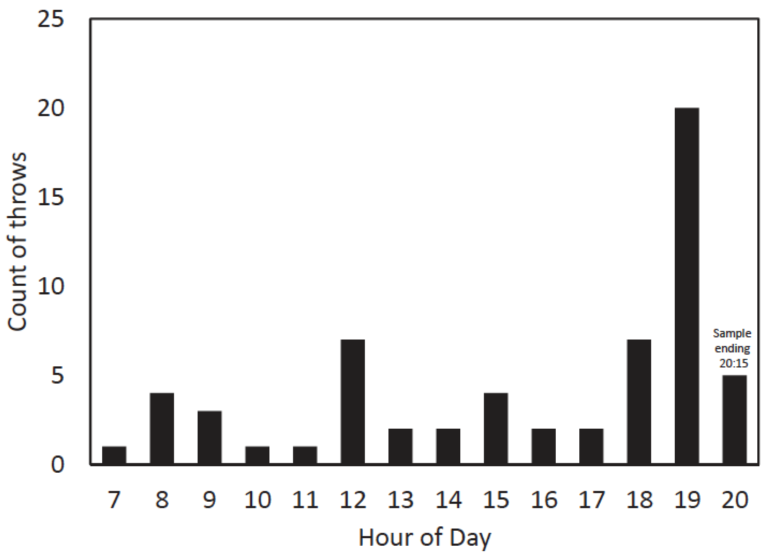

Figure S2: Throws by hour of occurrence during the day, 2015 data. On the day with 13.5 hours of sampling, throws occurred throughout daylight hours but increased in frequency around dusk

7. Details of hits

In 17 cases, as noted in the text, material thrown hit another octopus. In one additional case, a "hit" resulted as an octopus moved into the cloud after it was thrown. In two additional cases, material thrown hit a fish (minimally in one case). A large majority of the hits on other octopuses were due to the two throwers who threw most frequently in general: 5 hits from T1F and 9 from T23F. The other hits were single cases from three different individuals (probably one male and two female). A total of 15 of 17 hits were by behavioral females as identified by mating behavior, with one additional thrower a likely female due to other behaviors. The sex of the octopus hit by a throw could be assigned behaviorally in 13 cases out of 17. These comprised 8 hits on behavioral females (all sexes assigned by behaviors other than mating) and 5 on males (all assigned by mating, and probably all the most active male discussed in the main text).
